# Amikacin-eravacycline combination mediates the synergistic elimination of carbapenem-resistant pathogens via *in vitro* and *in vivo* metabolic reprogramming

**DOI:** 10.1371/journal.ppat.1013938

**Published:** 2026-02-10

**Authors:** Xiaoli Yang, Yili Chen, Jinmei Yang, Jiaying Lei, Tinghua Liu, Yougang Mai, Xikang Tang

**Affiliations:** 1 Department of Pediatrics, Sun Yat-sen Memorial Hospital, Sun Yat-sen University, Guangzhou, China; 2 Department of Laboratory Medicine, The First Affiliated Hospital of Sun Yat-sen University, Guangzhou, China; 3 Microbiology Laboratory, Department of Clinical Laboratory, Yantai Affiliated Hospital of Binzhou Medical University, Yantai, China; VIT University: Vellore Institute of Technology, INDIA

## Abstract

Carbapenem-resistant (CR) organisms (CRO) have been identified as critical priority pathogens, emphasizing the urgent need for novel therapeutic strategies. Combination therapy emerges as a promising approach to address multidrug-resistant bacterial infections. Here we demonstrate that eravacycline (ERV), in combination with amikacin (AMK), effectively eliminates a panel of clinically isolated CR *Escherichia coli*, CR *Klebsiella pneumoniae*, and CR *Acinetobacter baumannii*. Mechanistically, the AMK-ERV combination enhances bacterial oxidative phosphorylation, leading to an accumulation of reactive oxygen species, which induce oxidative stress and accelerate bacterial cell death. Notably, this combination significantly improves survival rates in mouse models of intra-abdominal infection, demonstrating efficacy against infections induced by CR pathogens. Furthermore, serum metabolomics reveals that the AMK-ERV combination upregulates metabolic pathways of lipids and amino acids. Interestingly, the amino acid methionine significantly enhances the antibacterial activity of ERV against CR pathogens both *in vitro* and *in vivo*. Our findings underscore the potential of repurposing AMK in combination with ERV to combat CR pathogens and propose a novel strategy for controlling these infections through the combination of antibiotics with specific metabolites such as methionine.

## Author summary

Carbapenem-resistant (CR) bacterial infections are a growing threat to global health and are becoming increasingly difficult to treat with existing drugs. Instead of developing new antibiotics, we explored more effective strategies using currently available medicines. We found that combining eravacycline (ERV), a recently approved antibiotic, with amikacin (AMK), a conventional and widely used drug, effectively eradicates multiple clinical isolates of CR pathogens. This combination disrupts bacterial energy metabolism and triggers overwhelming oxidative stress that leads to bacterial cell death. Unexpectedly, serum metabolomic analysis identified methionine, a natural amino acid, as a key factor that further enhances the activity of ERV. In a mouse model of intra-abdominal infection, this combination significantly reduced bacterial burden and improved survival. Mechanistically, this effect operates independently of the proton motive force, distinguishing it from the potentiation mechanism of tigecycline. Our findings highlight bacterial metabolism as a critical contributor to antibiotic efficacy and suggest that combining existing antibiotics with specific metabolic factors represents a practical therapeutic strategy against drug-resistant infections.

## Introduction

Antimicrobial resistance (AMR) has emerged as a critical global health crisis, recognized by the WHO as one of the top 10 threats to humanity, with profound implications across the One Health continuum [1–3]. Recent modeling from the Lancet projects 1.91 million direct AMR-attributable deaths and 8.22 million AMR-associated fatalities in 2050 if current trends persist [4]. While novel β-lactam/β-lactamase inhibitor combinations have expanded therapeutic options, the relentless spread of carbapenem-resistant (CR) organisms (CRO) presents unique therapeutic challenges [5]. Among Gram-negative bacteria, the annual death toll attributable to carbapenem resistance increased by 89,200 from 1990 to 2021, surpassing all other antibiotic classes over that period [4]. Urgent action is imperative to control CRO.

Currently, antibiotics remain the mainstay therapeutic intervention against bacterial infections. However, the emergence of resistance mechanisms has substantially compromised the clinical efficacy of existing drugs. Compounding this challenge, the protracted development cycle for novel antibiotics demonstrably lags behind the accelerated evolutionary trajectory of resistant strain emergence [6,7]. Moreover, non-standard use of new antibiotics may select for new resistant phenotypes, thereby trapping us in a vicious circle of constantly developing novel drugs to combat drug-resistant bacteria [8,9]. Numerous studies have highlighted that combination therapies, in which two or more antibiotics are strategically combined, are capable of yielding synergistic effects to effectively eradicate multidrug-resistant bacteria. Simultaneously, this multi-targeting approach can significantly lower the risk of new drug resistance mutations [10–12]. In this context, combination therapy presents itself as a more practical and effective solution for combating CRO.

Eravacycline (ERV), the first fully synthetic fluorocycline antibiotic, demonstrates broad-spectrum activity through ribosomal targeting [13]. Recent studies have shown *in vitro* synergistic effects between ERV and β-lactams or aminoglycosides against CRO, highlighting the therapeutic potential of ERV-based combination regimens for managing CRO infections [14–18]. While these studies have not yet investigated the mechanisms underlying the synergy, such investigations could facilitate the identification of drug-specific response biomarkers, offering novel therapeutic targets for combating CRO infections.

In this study, we systematically evaluated the synergy between ERV and traditional antibiotics. We found that the combination of amikacin (AMK) and ERV exhibited potent bactericidal activity against a panel of clinical isolates of CR *Escherichia coli* (CREC), CR *Klebsiella pneumoniae* (CRKP), and CR *Acinetobacter baumannii* (CRAB). In mouse models of intra-abdominal infection (IAI), this combination significantly improved survival rates. Mechanistic investigations revealed that the AMK-ERV combination activated oxidative phosphorylation, inducing reactive oxygen species (ROS) overproduction that caused oxidative damage, thereby accelerating pathogen eradication. Metabolomics profiling of serum from infected mice identified distinct upregulation of amino acid and lipid metabolic pathways within the combination-treated cohorts. Importantly, methionine (Met) potentiated the efficacy of ERV in eradicating CR pathogens both *in vitro* and *in vivo*. This study characterized interspecies metabolic responses to antibiotic interventions across bacterial and mouse models, offering new insights for developing therapeutic strategies against CR bacterial infections.

## Results

### AMK-ERV combination effectively eliminates CR pathogens *in vitro* and *in vivo*

**Fig 1:**
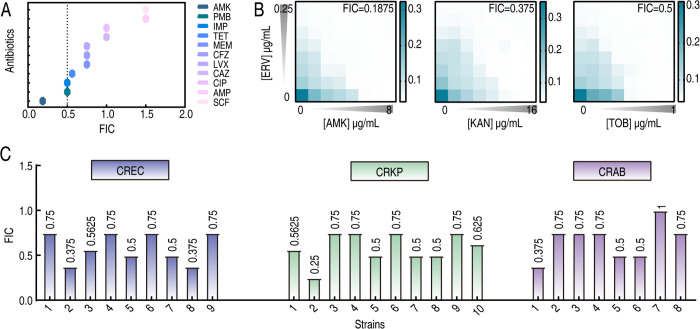
The synergistic effects of the AMK-ERV combination against CR pathogens *in vitro.* A. The efficacy of ERV in combination with various antibiotics against BW25113. B. The synergistic effects of ERV in combination with aminoglycoside antibiotics on BW25113. C. The effects of the AMK-ERV combination on CR pathogens.

To assess the antimicrobial synergism between ERV and traditional antibiotics, we employed a checkerboard assay using *E. coli* BW25113 as the reference strain. The results indicated indifferent interactions between ERV and either ampicillin (AMP) or cefoperazone-sulbactam (SCF). Additive effects were observed in combinations with tetracycline (TET), meropenem (MEM), cefazolin (CFZ), levofloxacin (LVX), ceftazidime (CAZ), and ciprofloxacin (CIP). Notably, ERV combined with AMK, polymyxin (PMB), or imipenem (IMP) exhibited significant synergistic activity, with the AMK-ERV pairing emerging as the most potent combination (FIC = 0.1875), reducing the MIC of AMK from 2 μg/mL to 0.25 μg/mL and the MIC of ERV from 0.125 μg/mL to 0.0078125 μg/mL (Fig 1A). Subsequent expansion to aminoglycosides showed significant synergy with kanamycin (KAN, FIC = 0.375) and tobramycin (TOB, FIC = 0.5; Fig 1B). Based on these findings, we focused on the AMK-ERV combination for extended evaluation against clinical CR isolates, including 9 CREC, 10 CRKP, and 8 CRAB (S1-S3 Tables). The regimen demonstrated synergy rates of 44.4% (4/9) in CREC, 40% (4/10) in CRKP, and 37.5% (3/8) in CRAB isolates, with additive effects noted in all remaining strains (Fig 1C and S4 Table).

**Fig 2:**
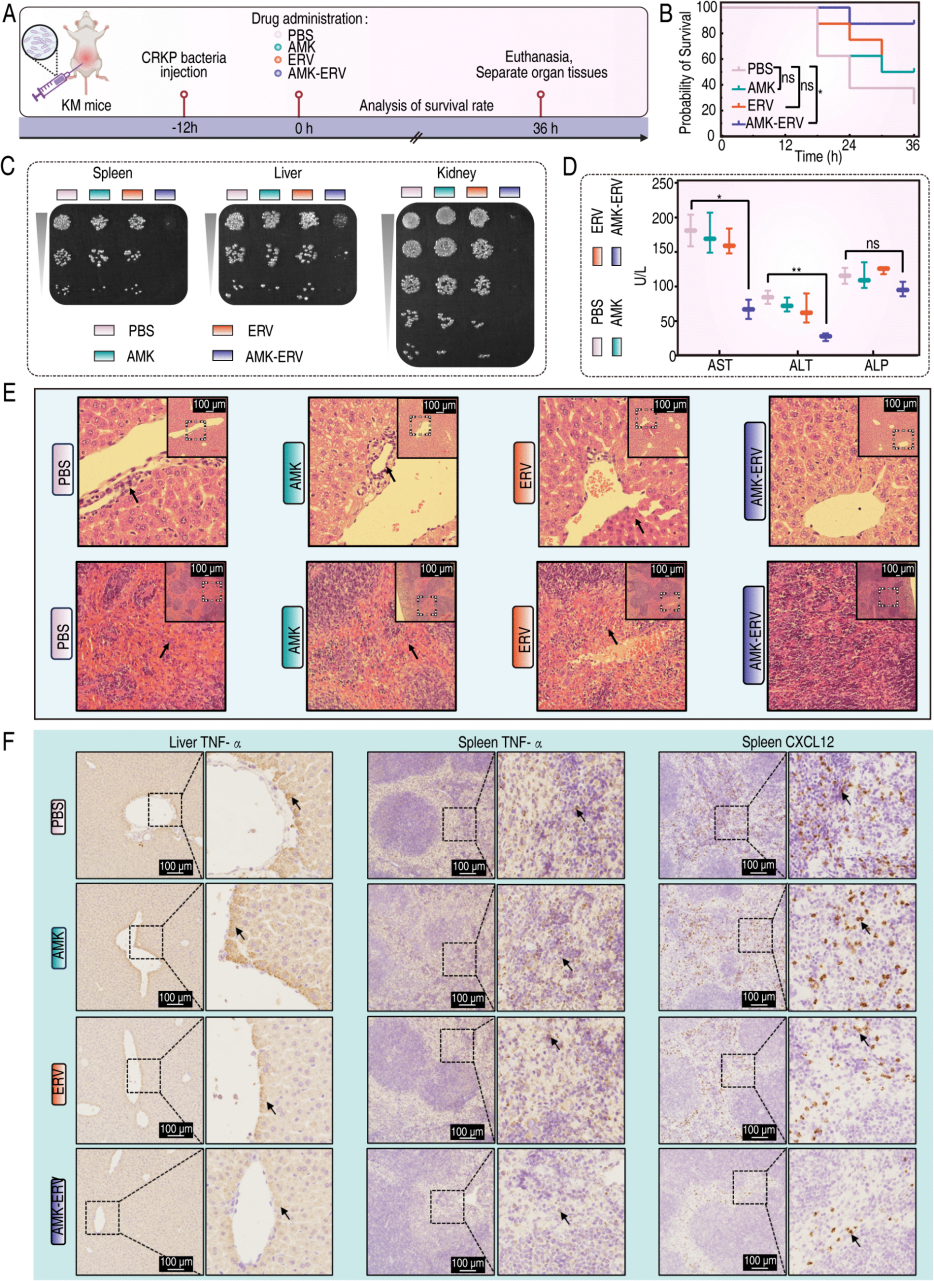
The synergistic effects of the AMK-ERV combination against CR pathogens *in vivo.* A. Schematic diagram illustrating the construction and treatment of mouse IAIs. Created in BioRender. Xiaoli, Y. (2025) https://BioRender.com/g99a722. B. Survival rates of mice in different treatment groups in the CRKP infection model (N = 8), analyzed using Log-rank test (ns *p* > 0.05; *p < 0.05). C. Determination of bacterial loads in the liver, spleen and kidney from mice following different treatment. D. Measurement of serum levels of liver enzymes as indicators of liver function. Comparisons between treatments and untreated controls were made using a Mann-Whitney test (ns *p* > 0.05; *p < 0.05; **p < 0.01). E. Pathological evaluation of the liver and spleen via H&E staining. F. Analysis of the expression levels of inflammatory factor in the liver and spleen using IHC.

The promising synergistic effects of AMK-ERV on CR pathogens observed *in vitro* prompted us to further evaluate its efficacy *in vivo*. Given ERV’s clinical indication for IAIs, we established a CRKP8-induced IAI mouse model (Fig 2A). At 36 hours post-treatment, untreated controls exhibited a mortality rate of 75%, compared to 50% in both AMK and ERV monotherapies. Notably, the combination therapy significantly reduced the mortality rate to 12.5% (Fig 2B). Quantitative culture analysis demonstrated a substantial decrease in bacterial burden within visceral organs (liver, kidneys, and spleen) following combination therapy, with minimal residual bacterial counts (Fig 2C). Liver enzyme analysis revealed significant reductions in aspartate aminotransferase (AST), alanine aminotransferase (ALT), and alkaline phosphatase (ALP) levels compared to those in the monotherapy groups (Fig 2D). Histopathological examination indicated that combination treatment markedly diminished hepatic inflammatory infiltration and alleviated splenic congestion compared to those in monotherapy groups (Fig 2E). Immunohistochemistry showed that liver sections from AMK-treated mice exhibited tumor necrosis factor-α (TNF-α) expression levels comparable to those in the control group. ERV monotherapy slightly reduced TNF-α levels, whereas the combination therapy significantly lowered this inflammatory marker. Spleen sections displayed elevated TNF-α and chemokine CXC ligand 12 (CXCL12) levels in both control and AMK groups. In contrast, these levels were moderately reduced in the ERV group and substantially diminished following the combination therapy (Fig 2F).

To evaluate the safety profile of the AMK-ERV combination at the dosage used in our efficacy studies, we conducted additional toxicity assessments in healthy mice using the same dosing regimen. Body weight changes and serum levels of AST, ALT, blood urea nitrogen (BUN), and creatinine (CREA) were monitored over a 72-hour period following administration. The results showed no significant loss in body weight and no significant abnormalities in liver or kidney function markers (S1A and S1B Fig), collectively indicating a lack of acute toxicity at the administered dosage.

Collectively, these results indicate that the AMK-ERV combination not only exhibits variable yet reproducible synergistic activity across different CR isolates *in vitro* and *in vivo* but also demonstrates a favorable safety profile at the administered dosage.

### AMK-ERV combination perturbs metabolic homeostasis and enhances oxidative phosphorylation

**Fig 3:**
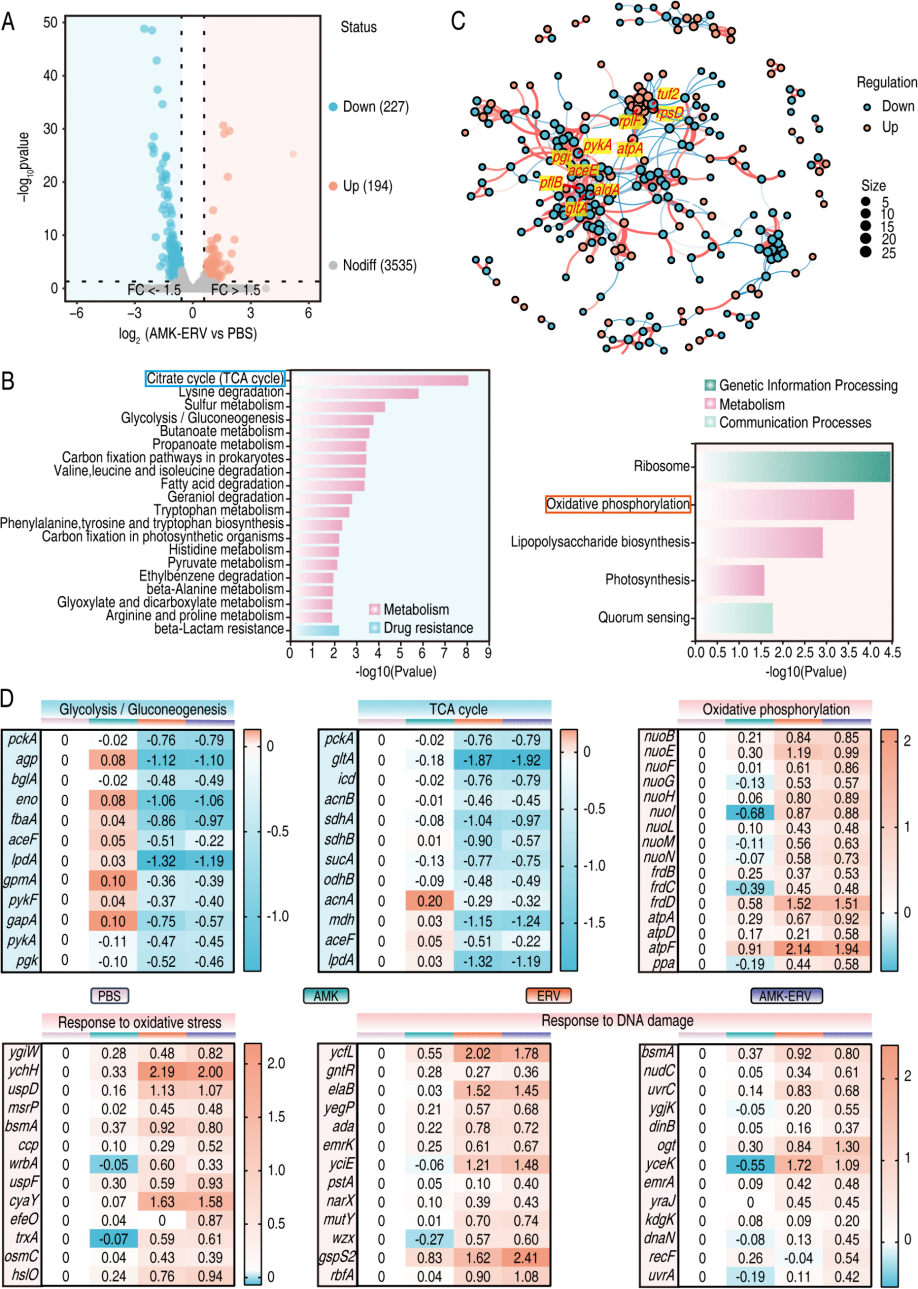
Transcriptomic responses of BW25113 exposed to combination treatment. A. Volcano plots showing the fold change and significance of genes. Fold changes are relative to control and are based on mean values of three independent experiments. B. KEGG pathway analyses for both up-regulated and down-regulated DEGs. C. PPI network demonstrating the hub genes. The dots represent genes (corresponding to proteins). The lines indicate interaction relationships; the size of the dots represents the number of interacting nodes. “up” indicates up-regulated genes, and “down” indicates down-regulated genes. D. The transcriptional changes of genes associated with oxidative stress compared with PBS group. The data, presented as log_2_ fold change, are derived from the mean values of three independent experiments.

To investigate the mechanism underlying observed synergy, we analyzed the transcriptome profile of the BW25113 strain following 4 hours of combination treatment. Hierarchical clustering analysis revealed extensive transcriptional changes induced by the AMK-ERV combination (S2 Fig). Using a threshold of ≥1.5-fold change and an adjusted *p* value <0.05, we identified 421 differentially expressed genes (DEGs), including 227 downregulated and 194 upregulated genes (Fig 3A). KEGG pathway analysis demonstrated distinct metabolic alterations; specifically, the downregulated DEGs were predominantly enriched in the TCA cycle (Fig 3B, left panel), while the upregulated DEGs exhibited significant enrichment in oxidative phosphorylation, secondary to ribosomal translation (Fig 3B, right panel). Protein-protein interaction (PPI) network analysis (interaction score >0.7) identified ten hub genes based on degree centrality: *gltA, aldA, pflB, pgi, aceE, pykA, tuf2, rplF, atpA*, and *rpsD* (Fig 3C). Functional annotation indicated that the downregulated cluster (*gltA, aldA, pflB, pgi, aceE, pykA*) encodes pivotal enzymes in central carbon metabolism. Ribosomal genes (*rplF* and *rpsD*) and oxidative phosphorylation-related *atpA* exhibited significant upregulation (S5 Table). Further analysis revealed that combination treatment markedly enhanced the expression of genes associated with complex I (*nuoB*, *nuoE*, *nuoF*, *nuoG*, *nuoH*, *nuoI*, *nuoL*, *nuoM*, and *nuoN*), complex II (*frdB*, *frdC*, and *frdD*), complex V (*atpA*, *atpD*, and *atpF*), as well as inorganic pyrophosphatase (*ppa*), all of which are involved in oxidative phosphorylation. Simultaneously, genes encoding enzymes in glycolysis/gluconeogenesis and the TCA cycle were downregulated. Notably, genes involved in oxidative stress response and DNA repair were upregulated (Fig 3D). These transcriptomic findings suggest a potential enhancement of oxidative phosphorylation. To determine whether these transcriptional changes led to a functional enhancement of oxidative phosphorylation, we measured intracellular ATP levels and the NADH/NAD^+^ ratio. The results confirmed that the combination therapy significantly increased ATP content and decreased the NADH/NAD^+^ ratio compared to the untreated control or either monotherapy (S3A and S3B Fig), indicating enhanced electron transport chain activity. Collectively, these data suggest that the AMK-ERV combination disrupts central carbon metabolism while potentiating oxidative phosphorylation, which we hypothesized would drive ROS overproduction and thereby enhance antimicrobial efficacy.

### Oxidative phosphorylation-driven ROS accumulation accelerates bacterial death

**Fig 4:**
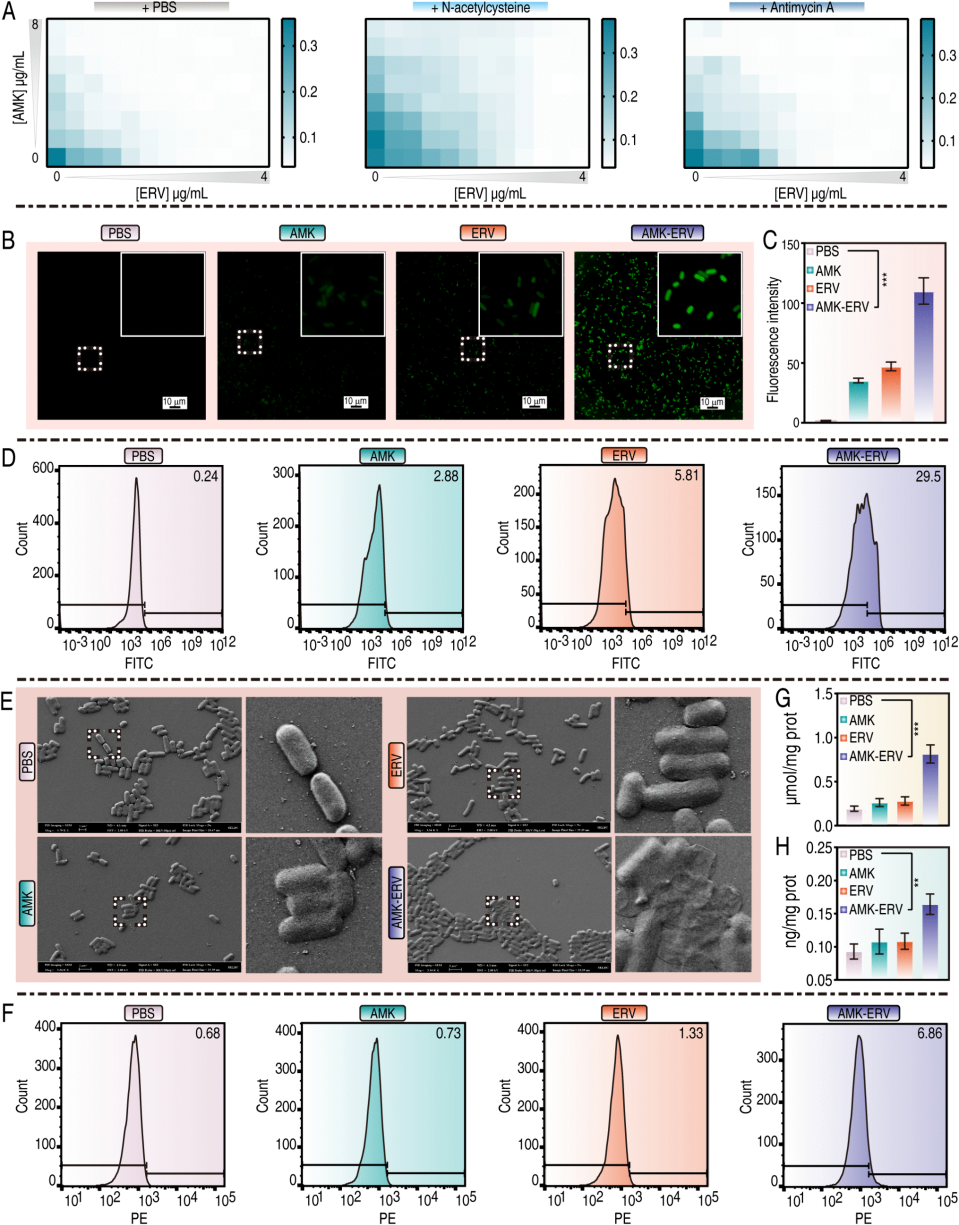
ROS mechanism. A. The influences of N-acetylcysteine and antimycin A on the synergistic bactericidal efficiency of AMK-ERV combination. B. Fluorescence microscopy visualizes DCF fluorescence to assess ROS levels. C. A bar graph showing DCF fluorescence intensity across four experimental groups. D. Flow cytometry quantifies ROS levels. E. SEM examines bacterial morphology. F. PI staining showing membrane integrity. G. Measurement of MDA content indicates lipid peroxidation. H. Determination of 8-OHdG concentrations reflects nucleic acid damage. All bar graphs display means ± SEM, with comparisons between treatments and untreated controls made using a Mann-Whitney test (**p < 0.01, ***p < 0.001).

Thus, we conducted further investigations into ROS. As shown in Fig 4A, N-acetylcysteine, a ROS scavenger, significantly reduced BW25113 susceptibility to both antibiotics and abolished the synergistic effects of their combination. A similar reduction was observed with antimycin A, an electron transport chain (ETC) inhibitor, suggesting that ETC-dependent ROS generation mediated this synergy. Fluorescence imaging using 2’,7’-dichlorofluorescein diacetate (DCFH-DA) revealed progressive ROS accumulation across treatment groups: control (1.5), AMK (35.4), ERV (47.1), and combination (110.1) (Fig 4B and 4C). Flow cytometric quantification of DCFH-DA positive cells corroborated these results: control (0.24%), AMK (2.88%), ERV (5.81%), and combination (29.5%) (Fig 4D). Scanning electron microscopy (SEM) demonstrated progressive ultrastructural damage: untreated controls maintained intact rod-shaped morphology, monotherapies caused mild collapse, while combination treatment resulted in severe cell wall collapse, with complete loss of native morphology and cellular rupture (Fig 4E). Propidium iodide (PI) staining showed minimal red fluorescence in the control and monotherapy groups, with increased signals in bacteria treated with the combination (Fig 4F). Lipid peroxidation analysis revealed significantly elevated malondialdehyde (MDA) levels in combination-treated bacteria compared to those treated with monotherapies (Fig 4G). Transcriptomic analysis identified significant upregulation of DNA repair genes in the combination group. Subsequent ELISA quantification confirmed elevated levels of 8-hydroxy-2′-deoxyguanosine (8-OHdG), a marker of oxidative DNA damage (Fig 4H). These results establish a self-amplifying oxidative damage cascade: hyperactivation of the ETC generates lethal ROS levels, inducing lipid peroxidation, oxidative DNA lesions, and membrane damage. Collectively, these effects overwhelm bacterial redox homeostasis, leading to accelerated bacterial death.

### AMK-ERV combination enhances amino acid and lipid metabolism in mouse serum

**Fig 5:**
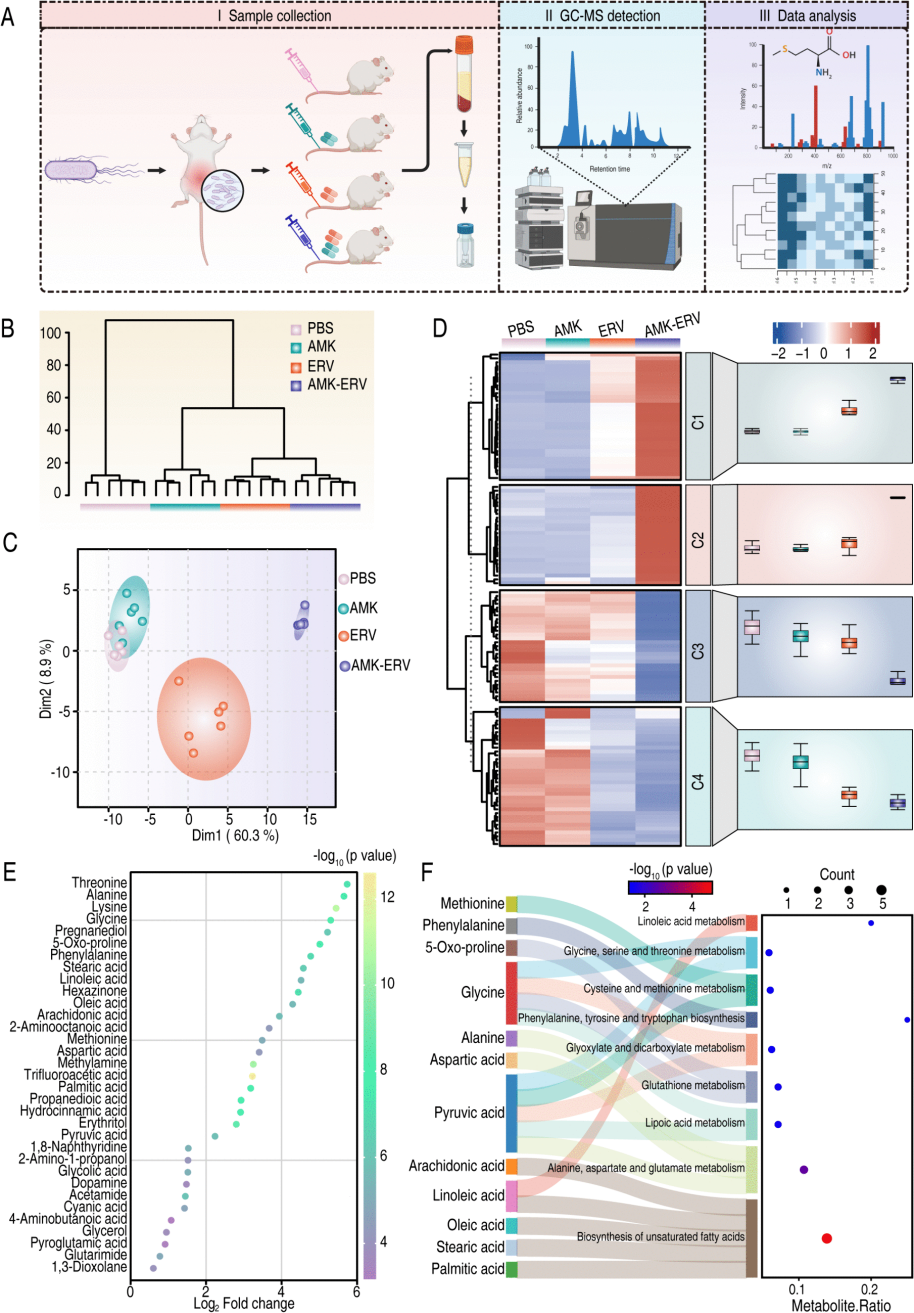
Metabolomics analysis of mouse serum. A. Identification of key serum metabolites through a comparative metabolomics approach. Created in BioRender. Xiaoli, Y. (2025) https://BioRender.com/g99a722. B. Hierarchical clustering dendrogram illustrating the similarity among samples. Six independent experiments are shown as replicates. C. PCA demonstrating the aggregation and dispersion of samples. Each point represents an individual sample, with distinct colors indicating different groups. D. Heatmap combined with trend plot depicting abundance changes of metabolites across four experimental groups, presented as mean values of six independent experiments. E. Variations in the abundance of differential metabolites. F. Pathway enrichment analysis of key metabolites. The size of dot for each pathway represents counts of enriched metabolites, and the color indicates significance.

We further investigated the serum metabolic profiles in CRKP8-infected mice with IAIs at 12 hours post-antibiotic interventions (Fig 5A). The experimental design comprised four groups: untreated controls, AMK monotherapy, ERV monotherapy, and AMK-ERV combination therapy, yielding 24 datasets. Hierarchical clustering revealed treatment-specific metabolic characteristics, with combination therapy specimens forming a distinct phenotypic cluster (Fig 5B). Principal component analysis (PCA) confirmed unique metabolic alterations in combination-treated mice (Fig 5C). Comparative analysis using the Mann-Whitney U test identified 124 differentially abundant metabolites between combination therapy and controls (*p* < 0.05). Mfuzz clustering delineated four distinct changing patterns (C1-C4). Although aminoglycosides such as AMK are effective synergistic partners, their clinical use is limited by well-recognized nephrotoxicity. Therefore, we explored whether metabolic supplementation could provide a non-antibiotic strategy to potentiate ERV while avoiding aminoglycoside-associated toxicity. Consequently, we identified a C1 metabolite cluster comprising 33 metabolites whose abundances were increased in the ERV group and further elevated in the combination group (Fig 5D). Among these, 22 metabolites exhibited marked changes (log2FC ≥ 2; Fig 5E). Pathway enrichment analysis using MetaboAnalyst mapped 12 of these metabolites to nine metabolic pathways. Specifically, six amino acids, including Met, phenylalanine (Phe), 5-oxo-proline, glycine (Gly), alanine (Ala) and aspartic acid (Asp), were associated with seven pathways, such as alanine/aspartate/glutamate metabolism, lipoic acid metabolism, glutathione metabolism, glyoxylate/dicarboxylate metabolism, phenylalanine/tyrosine/tryptophan biosynthesis, cysteine/methionine metabolism, and serine/threonine metabolism. In addition, five fatty acids (arachidonic acid, linoleic acid, oleic acid, stearic acid, and palmitic acid) were involved in the biosynthesis of unsaturated fatty acids and linoleic acid metabolism (Fig 5F). These metabolomic changes were associated with therapeutic improvement following combination treatment and were subsequently used to guide targeted functional validation experiments.

### Met improves ERV’s bactericidal efficacy against CR pathogens *in vitro* and *in vivo*

**Fig 6:**
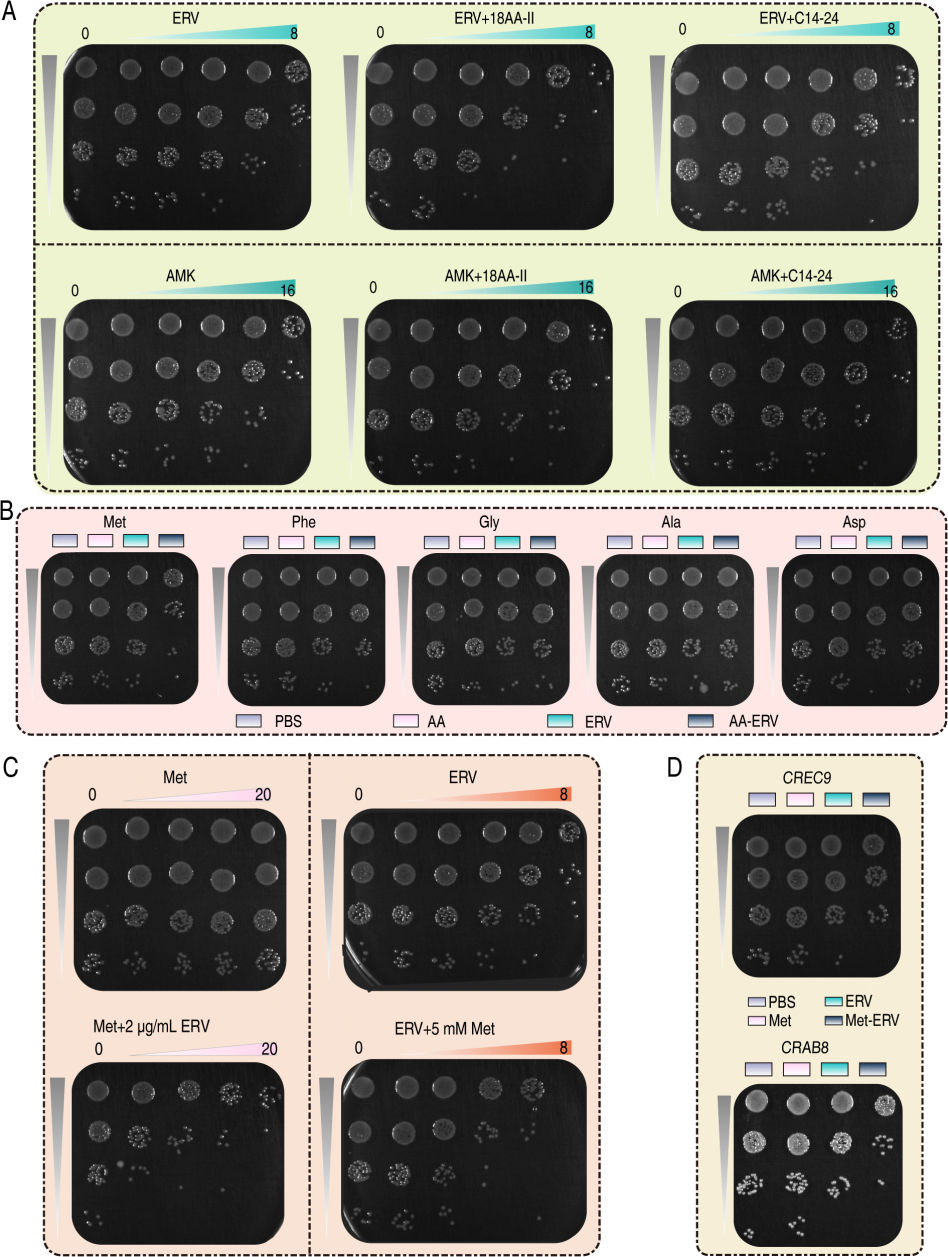
*In vitro* bactericidal efficacy of Met in combination with ERV. A. The effects of compound amino acids and fat emulsions on the bactericidal efficacy of AMK or ERV. B. The effect of amino acids on the elimination of CRKP8 by ERV. C. The bactericidal activity of Met in combination with ERV demonstrating concentration dependence. D. Met-ERV combination demonstrating synergistic activity against CRO.

To facilitate clinical translation, we subsequently utilized clinically available compound amino acid (18AA-II) and lipid emulsion (C14-24) as adjuvants to evaluate the efficacy of amino acids and lipids in enhancing the bactericidal activity of ERV. The results showed that 18AA-II significantly potentiated ERV’s bactericidal activity against CRKP8, while C14-24 did not exhibit such an effect. At an ERV concentration of 2 μg/mL, coadministration of 18AA-II reduced CRKP8 survival from approximately 50% to 5%. Doubling the ERV concentration (4 μg/mL) in combination with 18AA-II resulted in a 20-fold increase in efficacy, contrasting with the negligible potentiation of AMK by either supplement (Fig 6A). A screening of serum-enriched amino acids (Met, Phe, Gly, Ala, and Asp) identified Met as the sole synergist for ERV. 5 mM Met enhanced ERV-mediated killing of CRKP8 by 10-fold (Fig 6B). This potentiation was concentration-dependent: incremental increases in Met and ERV progressively improved bactericidal activity, achieving outcomes comparable to those observed with the coadministration of 18AA-II (Fig 6C). Importantly, Met-ERV synergy extended to other CR pathogens, such as CREC9, and CRAB8 (Fig 6D).

We next assessed whether the proton motive force (PMF), which has been shown to mediate Met-induced potentiation of the analog tigecycline, also contributes to its synergy with ERV [19]. Measurement of bacterial PMF confirmed that the inhibitor carbonyl cyanide 3-chlorophenylhydrazone (CCCP) effectively collapsed the PMF (S4A Fig). However, the potent bactericidal activity of the Met-ERV combination was not attenuated by CCCP co-treatment (S4B Fig), indicating a mechanism of action distinct from that of tigecycline.

**Fig 7:**
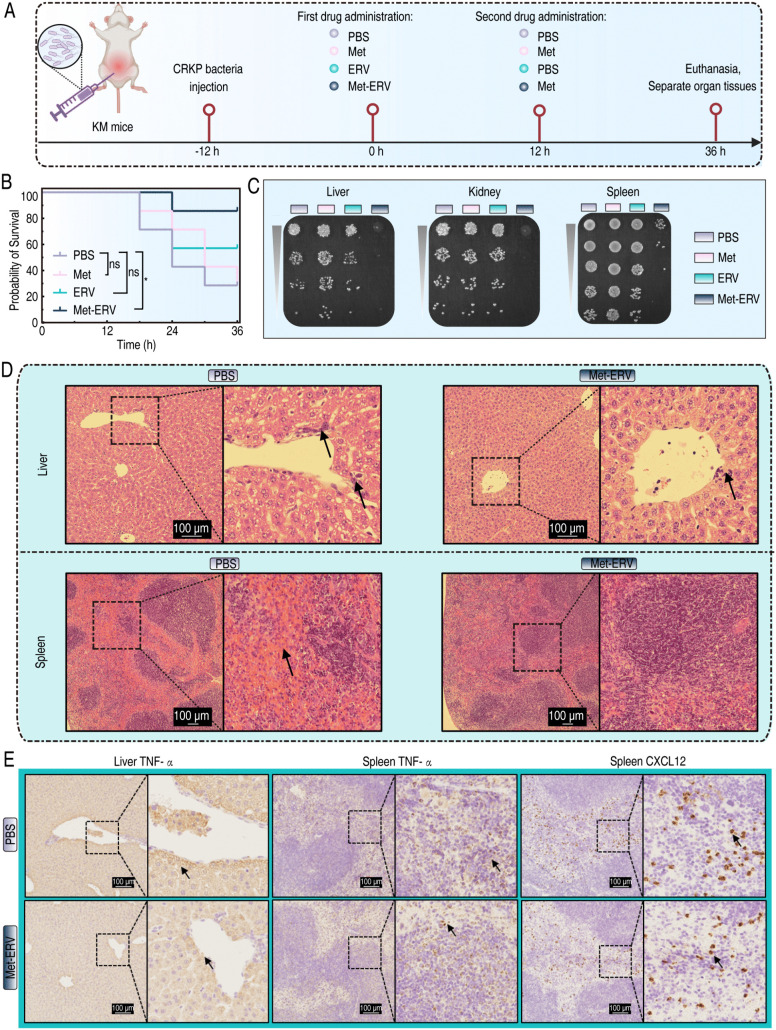
*In vivo* bactericidal efficacy of Met in combination with ERV. A. A schematic diagram showing the construction and treatment of mice with IAIs induced by CRKP8 pathogen. Created in BioRender. Xiaoli, Y. (2025) https://BioRender.com/g99a722. B. Survival rates of mice in different treatment groups (N = 7), analyzed using Log-rank test (ns *p* > 0.05; *p < 0.05). C. Measurement of bacterial loads in the liver, spleen, and kidney of mice following different treatment. D. H&E staining showing pathological changes in liver and spleen tissues of mice. E. IHC demonstrating the expression levels of pro-inflammatory factors within the liver and spleen.

To investigate the broader potential of this synergy beyond a single strain, we evaluated the Met-ERV combination *in vivo* using two additional clinically relevant CR isolates (CREC9 and CRAB8). The combination therapy also demonstrated significant synergistic effects in these models, indicating the extended efficacy observed *in vivo* (S5 Fig). We further delineated the therapeutic potential of the Met-ERV combination in a CRKP8-induced IAI model (Fig 7A), Met alone did not improve survival compared to controls (74% vs 74%). The ERV monotherapy group exhibited a mortality rate of 42.9%. Notably, the Met-ERV combination significantly reduced mortality to 14.3% (Fig 7B). Bacterial burden analysis revealed limited efficacy of ERV monotherapy, while Met alone paradoxically led to splenic bacterial proliferation. The combination therapy, however, demonstrated superior clearance in the liver, spleen, and kidneys (Fig 7C). Histopathological evaluation indicated that combination treatment alleviated hepatic inflammation and splenic congestion observed in the control group (Fig 7D). Immunohistochemical analysis further showed that combination therapy markedly reduced TNF-α levels in liver tissue, as well as TNF-α and CXCL12 levels in spleen tissues compared to those in control group (Fig 7E). These results suggest that the Met-ERV combination effectively eliminates CR pathogens both *in vitro* and *in vivo*.

## Discussion

Several current clinical consensus statements and guidelines recommend antibiotic combination therapy for CR bacterial infections [20–22]. ERV, a novel antibiotic approved in China in 2023, however, emerging surveillance data (2024) from the China Antimicrobial Surveillance Network (CHINET) reveal concerning resistance patterns, with 2.7% (n = 1,267) of *E. coli* and 6.1% (n = 1,267) of *K. pneumoniae* isolates displaying reduced ERV susceptibility [23]. If left unchecked, this will repeatedly enter a vicious cycle of drug resistance. Combination antibiotic therapy has been shown to effectively eradicate MDR pathogens, while delaying the emergence of resistance and reducing toxic side effects [10–12]. Therefore, it is essential to consider a combination therapy strategy for ERV. Recent studies have shown *in vitro* synergistic effects between ERV and β-lactams or aminoglycosides against CR bacteria [14–18]. In this study, we demonstrated that the AMK-ERV combination can effectively eliminate CR pathogens both *in vitro* and *in vivo*, including those with reduced susceptibility to ERV. Furthermore, we uncovered the metabolic interplay underlying this synergism in a bacterial system and a mouse model, which has not been previously reported.

A key finding of this study is that the AMK-ERV combination exerts synergistic effects through ROS-mediated mechanisms, highlighting redox homeostasis as a targetable vulnerability in CR pathogens. Antimicrobial agents primarily exert bactericidal effects via target-specific molecular interactions [24,25]. Both AMK and ERV inhibit prokaryotic protein synthesis by binding to the 30S ribosomal subunit [13,26]. The enhanced ribosomal biogenesis revealed in transcriptomic analysis may represent a compensatory feedback response elicited by the primary bactericidal mechanism. Importantly, transcriptomic analysis also uncovered profound perturbations in energy metabolism characterized by enhanced oxidative phosphorylation coupled with an impaired TCA cycle, which have not been reported before. The observed increase in intracellular ATP coupled with a decreased NADH/NAD^+^ ratio functionally validated that the combination therapy robustly enhances oxidative phosphorylation. During oxidative phosphorylation via the ETC, partial electron leakage generates ROS (O_2_^-^) through spontaneous oxygen reduction. Pharmacological inhibition using the antioxidant (N-acetylcysteine) and the oxidative phosphorylation inhibitor (antimycin A) substantially attenuated the synergistic effects of combination treatment, substantiating the oxidative phosphorylation-dependent, ROS-mediated synergistic bactericidal effects. Collins et al. reported that bactericidal antibiotics activate the ETC, leading to substantial ROS accumulation that mechanistically contributes to bacterial lethality [27]. Accumulating evidence indicates that the primary bactericidal actions of antibiotics induce metabolic reprogramming, thereby potentiating ROS overproduction. The excessive accumulation of ROS triggers oxidative stress cascades, which accelerate bacterial cell death [25,27–30]. Here, the precise metabolic circuitry linking ribosomal targeting (the primary effect) to oxidative phosphorylation potentiation (the secondary effect) requires further investigation. However, current primary drug targets exhibit well-characterized resistance mechanisms; consequently, the non-specific oxidative damage mediated by ROS holds considerable therapeutic potential [31]. Strategically amplifying bacterial oxidative stress by modulating redox homeostasis regulators may represent a promising therapeutic strategy against CR pathogen infections.

Another core finding of this study is the identification of Met as a potent enhancer of ERV’s efficacy against CR pathogens. This synergistic effect was consistently demonstrated both *in vitro* and *in vivo* across multiple clinically relevant strains (including CRKP8, CREC9 and CRAB8), suggesting a novel therapeutic avenue. Fang et al. demonstrated that Met enhances tigecycline efficacy against tet(X4)-positive pathogens by upregulating the PMF to promote antibiotic accumulation [19]. Intriguingly, our mechanistic exploration revealed that the synergy between Met and ERV operates through a PMF-independent pathway, thus indicating a distinct mechanism of action from that observed with tigecycline. Moreover, the observation of robust, concentration-dependent synergy *in vitro*, where pharmacokinetic (PK) variables are controlled, provides evidence for a direct pharmacodynamic (PD) interaction, wherein Met modulates the bacterial response to ERV.

Simultaneously, our approach to discovering Met underscores the value of host-focused serum metabolomics as a discovery platform. While comparative metabolomic profiling of antibiotic-sensitive and resistant bacterial strains has identified metabolites that reverse resistance [32,33], these findings have often demonstrated limited clinical translatability, possibly because bacterial culture conditions do not fully recapitulate the *in vivo* host environment. In contrast, metabolites identified from the host serum metabolome following successful treatment may possess higher clinical potential, as they function within a pathophysiologically relevant context. This approach is well-established in fields like oncology, where serum metabolite analysis has yielded clinically actionable biomarkers and therapeutic insights [34,35]. Guided by this rationale, we identified elevated Met levels in the serum of infected mice that responded to combination therapy. It is important to note that the elevated serum Met, while a promising correlate of treatment success, could also reflect systemic metabolic normalization following bacterial clearance. Furthermore, the observation that Met monotherapy increased the splenic bacterial burden *in vivo*, consistent with its growth-promoting effect *in vitro*, might be attributed to the spleen’s high vascularization, potentially leading to elevated local Met concentrations that fuel bacterial proliferation. This finding crucially underscores that the therapeutic benefit is strictly dependent on its co-administration with ERV. Although the present study provides evidence for a PD-based synergy, the potential for Met to influence the pharmacokinetic profile of ERV *in vivo* remains an open question. Therefore, future studies are warranted to fully elucidate the precise molecular mechanism of this PD interaction and to definitively assess potential PK interactions.

## Conclusion

**Fig 8:**
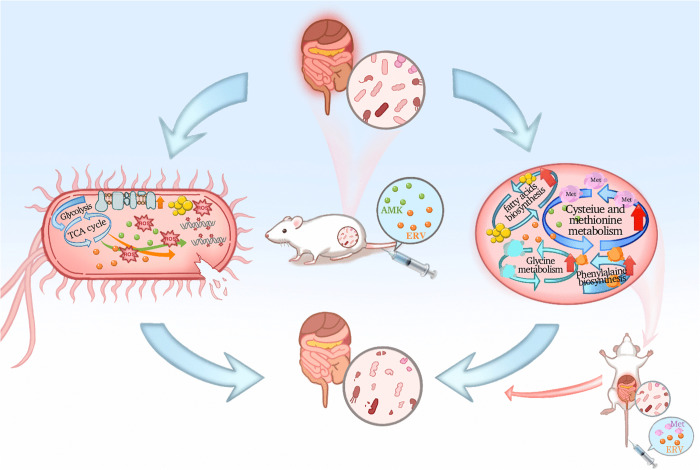
A proposed working model illustrates the mechanism underlying the AMK-ERV combination against CRO. The AMK-ERV combination activates oxidative phosphorylation, leading to an overproduction of ROS. The excessive accumulation of ROS induces oxidative stress cascades, resulting in peroxidative damage to lipids and nucleic acids, thereby accelerating bacterial cell death. Concomitantly, this combination therapy enhances lipid and amino acid metabolism in murine serum. Met is identified as a critical metabolite that significantly potentiates the antimicrobial activity of ERV against CRO.

In conclusion, this study validated the synergistic bactericidal activity of the AMK-ERV combination against CR clinical isolates through *in vitro* and *in vivo* experiments. Integrated multi-omics analyses revealed dual metabolic mechanisms, characterizing bacterial oxidative stress accumulation and host Met metabolism modulation, and identified the Met-ERV adjuvant strategy exhibiting effective eradication of tested CR isolates in an infection model (Fig 8). These findings enhance our understanding of the pathogen-host metabolic crosstalk underlying antimicrobial efficacy, offering valuable insights for developing innovative therapeutic strategies against CRO infections. Nevertheless, the unresolved mechanistic relationship between host metabolic reprogramming and enhanced antibiotic activity requires further investigation.

## Materials and methods

### Ethics statement

All experiments involving animals were conducted in accordance with the ethical policies and procedures approved by the Institutional Animal Care and Use Committee of Sun Yat-Sen University (SYSU-IACUC-2024–002064).

### Strains

The *E. coli* BW25113 strain was generously provided by South China Agricultural University. Clinical isolates were obtained from the collection at The First Affiliated Hospital of Sun Yat-sen University and the Yantai Affiliated Hospital of Binzhou Medical University.

### Antibiotics and chemicals

ERV was generously donated by Everest Medicines, while all other antibiotics, NAC, DCFH-DA, PI, glutaraldehyde, formaldehyde, pyridine, hydrochloric methoxyamine, and N-methyl-N-(trimethylsilyl) trifluoroacetamide were purchased from Sigma-Aldrich. Phosphate-buffered saline (PBS) was obtained from Guangzhou Jet Bio-Filtration Co., Ltd. Anti-CXCL12 Rabbit pAb (GB111624), Anti-TNF-alpha Rabbit pAb (GB11188) and HRP conjugated Goat Anti-Rabbit IgG (GB23303) were procured from Wuhan servicebio technology Co., Ltd. The lipid emulsion and compounded amino acids were sourced from Jilin Aodong Pharmaceutical Group Co., Ltd. and Chenxin Pharmaceutical Co., Ltd, respectively. Additional amino acids were procured from BBI Life Sciences Corporation.

### Media and growth conditions

Unless specified otherwise, all cells were cultured at 37°C with shaking at 200 rpm in their respective media. Overnight cultures were obtained by inoculating cells from frozen stock into fresh LB (hopebio). Exponential-phase cells were prepared by diluting overnight cultures 1:100 in fresh LB and incubating until OD _600 nm_ reached ~ 0.5. An additional sublethal concentration antibiotic was added to the cells, followed by 4 h of incubation before measuring relevant indicators. Stationary-phase cells were collected by cultivating cells in LB to OD _600 nm_ ~ 0.25-0.3, then re-inoculating these cultures at 1:1,000 dilution in fresh LB and incubating for 16 h.

### Combination susceptibility testing of antibiotics

The experiment employed the checkerboard assay [36]. Briefly, two antibiotics were separately added to the rows and columns of a 96-well plate, followed by serial twofold dilutions. Subsequently, exponential-phase bacteria were diluted at 1:40 in MH broth (hopebio) and introduced into each well, achieving a final concentration of 5 × 10^5 CFU/mL. After incubating at 37°C for 18 h, absorbance was measured at 600 nm using a Microplate Reader. The FIC index was calculated with the formula: FIC = (MIC_A_ combination/MIC_A_ monotherapy) + (MIC_B_ combination/MIC_B_ monotherapy). FIC ≤ 0.5, 0.5 < FIC ≤ 1, 1 < FIC ≤ 2, FIC > 2 indicate synergism, additive effect, indifference and antagonism, respectively [37].

### IAIs mouse model

All animal procedures are in accordance with the Guide for the Care and Use of Laboratory Animals (8th edition) [38]. For this experiment, we used KM female mice aged 6–8 weeks from the Laboratory Animal Center of Sun Yat-sen University. The stationary-phase cells were washed and suspended in PBS to a concentration of 10^8 CFU/mL. A 200 μL suspension was injected into the peritoneal cavity of each mouse. 12 h post-infection, the infected mice were randomly divided into four groups and treated with different drugs via tail vein injection. (1) AMK-ERV combination therapy: (I) PBS; (II) AMK (5 mg/kg); (III) ERV (5 mg/kg); (IV) AMK (5 mg/kg) combined with ERV (5 mg/kg) [39–41]. (2) Met-ERV combination therapy: (I) PBS; (II) Met (50 mg/kg); (III) ERV (5 mg/kg); (IV) Met (50 mg/kg) combined with ERV (5 mg/kg). 12 h later, mice were treated again: (I) PBS; (II) Met (50 mg/kg); (III) PBS; (IV) Met (50 mg/kg) [42,43]. Continuously monitored the survival status of each group of mice within 36 h following administration to assess the survival rate. After this period, the mice were anesthetized, and blood samples were collected through the posterior orbital venous plexus. Euthanasia was then performed via carbon dioxide inhalation using a gradual-fill method with a displacement rate of 30–70% of the chamber volume per minute. The CO_2_ flow was maintained for at least 1 min after respiratory arrest [44,45]. This was followed by isolation of the liver, kidneys, and spleen. Tissue samples were homogenized, diluted fivefold serially, and inoculated onto LB agar plates at 37°C for 16 h. The number of colonies was recorded to determine the bacterial load in the tissues. Additionally, organ tissues underwent histological analysis using hematoxylin-eosin (H&E) staining and IHC, with imaging conducted using a fluorescence microscope (Nikon Eclipse Ni-E). Serum was immediately shock-frozen after blood draw and stored at -80°C for further analysis.

### Pathological staining

H&E staining and immunohistochemical experiment reports are detailed in S1 Text.

### Transcriptional profiling

A complete description of the experimental method and transcriptome analysis flow is available in S2 Text.

### Metabolomics workflow

A complete description of the experimental method and analysis workflow is provided in S3 Text.

### Measurement of ROS

The cells treated with sub-lethal doses of antibiotics were washed and resuspended in PBS. Added DCFH-DA (10 μM) and incubated at 37°C in the dark for 30 min. After incubation, cells were washed three times with PBS to remove uninternalized probes. Directly observed the fluorescence using a fluorescence microscope and analyzed the intensity with ImageJ software [46]. Additionally, DCF fluorescence was detected through the FITC channel of a flow cytometer (CytoFLEX) to assess ROS levels and evaluate oxidative stress [47].

### Observation of bacterial morphology

Added 2.5% glutaraldehyde to the cells treated with sublethal concentrations of antibiotics, and incubated them at room temperature for 30 min. Sequentially dehydrate the cells using ethanol at gradient concentrations (30%, 50%, 70%, 80%, and 95%), with each step lasting 5 minutes. Resuspend the bacteria in a 95% ethanol solution and apply 5 μL of the suspension onto a glass slide. After drying, a gold film layer was deposited on the surface [48]. Observed and photographed the sample using a SEM (Carl Zeiss Crossbeam 550).

### Detection of bacterial membrane integrity

Cells treated with sublethal concentrations of antibiotic were washed and resuspended in PBS. PI dye was added and incubated in the dark at 37°C for 15 min. After incubation, cells were washed three times with PBS to remove uninternalized dye. The fluorescence intensity of PI was detected through the PE channel by using a flow cytometer to evaluate the integrity of bacterial membranes [49].

### Detection of MDA and 8-OhdG levels

MDA levels and 8-OhdG levels were quantified using the Lipid Peroxidation MDA Assay Kit (Beyotime) and the 8-OHdG ELISA Kit (Jingmei), respectively.

### Bactericidal test

This experiment was performed as previously described [50,51]. The growth and survival of untreated stationary-phase cells were compared to cultures treated with antibiotic alone (AMK or ERV), metabolite alone (0.85% 18 AA-II, 2% C14-24, 5 mM Met, 5 mM Phe, 5 mM Gly, 5 mM Ala, 5 mM Asp), or a combination of antibiotic and metabolite for 6 h. Stationary-phase cells were washed and resuspended in PBS to a concentration of 10^7 CFU/mL. This suspension was then aliquoted into multiple test tubes, with 5 mL per tube. Individual additions of metabolites and antibiotics were added to each tube, followed by incubation at 37°C with shaking at 200 rpm for 6 h. At specified intervals, 100 μL samples were taken for tenfold serial dilution and plated on LB agar to count CFU/mL. Statistical analysis was performed on dilutions yielding between 20 and 100 colonies. The cell survival percentage was determined by comparing the CFU/mL values of the experimental groups against those of the control group.

### Measurement of ATP levels

The experimental method is described in S4 Text.

### Measurement of NADH/NAD^+^ ratio

Details of the experimental procedure are provided in S5 Text.

### PMF determination

The methodology is detailed in S6 Text.

## Supporting information

S1 Fig Assessment of acute toxicity. A. Body weight changes in healthy mice after administration across the four experimental groups. B. Serum biochemistry analysis of liver and kidney function markers at 72 h. Data are presented as mean ± SEM. No significant abnormalities were detected.
(TIF)

S2 Fig Hierarchical clustering of gene expression levels for the BW25113 strain subjected to combined antibiotic treatment. The expression levels are represented by a color gradient ranging from blue (low expression) to red (high expression).
(TIF)

S3 Fig Assessment of oxidative phosphorylation activity. A. Measurement of intracellular ATP levels following antibiotic treatment. B. Measurement of the NADH/NAD+ ratio. Data are presented as mean ± SEM. **p < 0.01.
(TIF)

S4 Fig The role of the PMF in Met-ERV synergy. A. Measurement of the bacterial PMF following treatment with CCCP. B. Effects of CCCP on the bactericidal efficacy of the Met-ERV combination.
(TIF)

S5 Fig Survival rates of mice in CREC and CRAB infection models. Statistical significance was determined by the Log-rank test (ns *p* > 0.05; *p < 0.05).
(TIF)

S1 Table The antibiotic susceptibility of CREC clinical isolates. (DOCX)

S2 Table The antibiotic susceptibility of CRKP clinical isolates. (DOCX)

S3 Table The antibiotic susceptibility of CRAB clinical isolates. (DOCX)

S4 Table MICs of AMK and ERV alone and in combination against tested CR strains. (DOCX)

S5 Table The functional annotation of hub genes. (DOCX)

S1 Text Pathological staining. (DOCX)

S2 Text Transcriptional profile. (DOCX)

S3 Text Metabolomics workflow. (DOCX)

S4 Text Measurement of ATP levels. (DOCX)

S5 Text Measurement of NADH/NAD^+^ ratio. (DOCX)

S6 Text PMF determination. (DOCX)
