# Supplementary material for: Amikacin-eravacycline combination mediates the synergistic elimination of carbapenem-resistant pathogens via in vitro and in vivo metabolic reprogramming
Source: PLoS Pathog. 2026 Feb 10;22(2):e1013938. doi: 10.1371/journal.ppat.1013938 (PMC12890146; doi:10.1371/journal.ppat.1013938)
Supplement: S1 Table — (DOCX) [file ppat.1013938.s007.docx]

**S2 Table The antibiotic susceptibility of CRKP clinical isolates**

|  | CRKP1 | CRKP2 | CRKP3 | CRKP4 | CRKP5 | CRKP6 | CRKP7 | CRKP8 | CRKP9 | CRKP 10 |
| --- | --- | --- | --- | --- | --- | --- | --- | --- | --- | --- |
| AMP | / | / | / | / | / | / | / | **S** | / | / |
| CAZ | R | R | R | R | R | R | R | **R** | R | R |
| TIM | R | R | R | R | R | R | R | R | R | R |
| TOB | S | R | S | S | S | I | S | R | I | S |
| TZP | R | R | R | R | R | R | R | R | R | R |
| AMK | S | R | S | S | S | S | S | **S** | S | S |
| ETP | R | R | R | R | R | R | R | R | R | R |
| MEM | R | R | R | R | R | R | R | **R** | R | R |
| AMC | R | R | R | R | R | R | R | R | R | R |
| PMB | S | S | S | S | S | S | S | S | S | S |
| LVX | R | R | R | R | R | R | R | **R** | R | R |
| TGC | S | S | S | S | / | S | S | R | S | S |
| SCF | R | R | R | R | R | R | R | R | R | R |
| CIP | R | R | R | R | R | R | R | R | R | R |
| SAM | R | R | R | R | R | R | R | **S** | R | R |
| FAZ | R | R | R | R | R | R | R | R | R | R |
| FEP | R | R | R | R | R | R | R | R | R | R |
| FOX | R | R | R | R | R | R | R | R | R | R |
| GEN | S | R | S | S | S | R | R | **/** | R | R |
| IPM | R | R | R | R | R | R | R | **I** | R | R |
| ATM | R | R | R | R | R | R | R | R | R | R |
| MIN | I | R | S | R | R | I | R | R | R | R |
| CHL | R | R | S | R | / | R | R | / | I | I |
| CRO | R | R | R | R | R | R | R | **R** | R | R |
| CTX | R | R | R | R | R | R | R | R | R | R |
| ERV | 0.5 | 1 | 0.125 | 0.5 | 1 | 0.5 | 1 | 2 | 2 | 1 |

**a.** AMP: Ampicillin, CAZ: Ceftazidime, TIM: Ticarcillin/Clavulanic acid, TOB: Tobramycin, TZP: Piperacillin/Tazobactam, AMK: Amikacin, ETP: Ertapenem, MEM: Meropenem, AMC: Amoxicillin/Clavulanic acid, PMB: Polymyxin B, LVX: Levofloxacin, TGC: Tigecycline, SCF: Cefoperazone/Sulbactam, CIP: Ciprofloxacin, SAM: Ampicillin/Sulbactam, FAZ: Cefazolin, FEP: Cefepime, FOX: Cefoxitin, GEN: Gentamicin, IPM: Imipenem, ATM: Aztreonam, MIN: Minocycline, CHL: Chloramphenicol; CRO: Cefuroxime, CTX: Cefotaxime, ERV: Eravacycline.

**b.** The breakpoints for eravacycline resistance have not yet been determined. ECAST released the susceptibility breakpoint for *Klebsiella pneumoniae* to this antibiotic, which is ≤1 μg/mL.

**c.** /: Non, S: Susceptible, I: Intermediate, R: Resistant.
