# Supplementary material for: Amikacin-eravacycline combination mediates the synergistic elimination of carbapenem-resistant pathogens via in vitro and in vivo metabolic reprogramming
Source: PLoS Pathog. 2026 Feb 10;22(2):e1013938. doi: 10.1371/journal.ppat.1013938 (PMC12890146; doi:10.1371/journal.ppat.1013938)
Supplement: S3 Table — (DOCX) [file ppat.1013938.s009.docx]

**S4 Table MICs of AMK and ERV alone and in combination against tested CR strains**

| **Strain** | **Antibiotic Combination** | **MIC Alone**  **(μg/mL)** | **MIC in Combination**  **(μg/mL)** | **FIC Index** | **Interpretation** |
| --- | --- | --- | --- | --- | --- |
| CREC1 | AMK | 4 | 2 | 0.75 | Additive effect |
|  | ERV | 0.125 | 0.03125 |  |  |
| CREC2 | AMK | 0.5 | 0.125 | 0.375 | Synergism |
|  | ERV | 0.0625 | 0.0078125 |  |  |
| CREC3 | AMK | 2 | 1 | 0.5625 | Additive effect |
|  | ERV | 0.25 | 0.015625 |  |  |
| CREC4 | AMK | 2 | 0.5 | 0.75 | Additive effect |
|  | ERV | 0.25 | 0.125 |  |  |
| CREC5 | AMK | 128 | 32 | 0.5 | Synergism |
|  | ERV | 1 | 0.25 |  |  |
| CREC6 | AMK | 4 | 2 | 0.75 | Additive effect |
|  | ERV | 0.25 | 0.0625 |  |  |
| CREC7 | AMK | 1 | 0.25 | 0.5 | Synergism |
|  | ERV | 0.125 | 0.03125 |  |  |
| CREC8 | AMK | 2 | 0.5 | 0.375 | Synergism |
|  | ERV | 0.25 | 0.03125 |  |  |
| CREC9 | AMK | 32 | 16 | 0.75 | Additive effect |
|  | ERV | 0.125 | 0.03125 |  |  |
| CRKP1 | AMK | 0.5 | 0.25 | 0.5625 | Additive effect |
|  | ERV | 0.5 | 0.03125 |  |  |
| CRKP2 | AMK | 128 | 16 | 0.25 | Synergism |
|  | ERV | 1 | 0.125 |  |  |
| CRKP3 | AMK | 4 | 2 | 0.75 | Additive effect |
|  | ERV | 0.125 | 0.3125 |  |  |
| CRKP4 | AMK | 8 | 2 | 0.75 | Additive effect |
|  | ERV | 0.5 | 0.25 |  |  |
| CRKP5 | AMK | 16 | 4 | 0.5 | Synergism |
|  | ERV | 1 | 0.25 |  |  |
| CRKP6 | AMK | 4 | 2 | 0.75 | Additive effect |
|  | ERV | 0.5 | 0.125 |  |  |
| CRKP7 | AMK | 0.25 | 0.0625 | 0.5 | Synergism |
|  | ERV | 1 | 0.25 |  |  |
| CRKP8 | AMK | 4 | 1 | 0.5 | Synergism |
|  | ERV | 2 | 0.5 |  |  |
| CRKP9 | AMK | 8 | 4 | 0.75 | Additive effect |
|  | ERV | 2 | 0.5 |  |  |
| CRKP10 | AMK | 2 | 0.25 | 0.625 | Additive effect |
|  | ERV | 1 | 0.5 |  |  |
| CRAB1 | AMK | 2 | 0.5 | 0.375 | Synergism |
|  | ERV | 0.0625 | 0.0078125 |  |  |
| CRAB2 | AMK | 1 | 0.5 | 0.75 | Additive effect |
|  | ERV | 0.0625 | 0.015625 |  |  |
| CRAB3 | AMK | 0.5 | 0.25 | 0.75 | Additive effect |
|  | ERV | 0.0625 | 0.015625 |  |  |
| CRAB4 | AMK | 16 | 4 | 0.75 | Additive effect |
|  | ERV | 0.0625 | 0.03125 |  |  |
| CRAB5 | AMK | 8 | 2 | 0.5 | Synergism |
|  | ERV | 0.0625 | 0.015625 |  |  |
| CRAB6 | AMK | 2 | 0.5 | 0.5 | Synergism |
|  | ERV | 0.0625 | 0.015625 |  |  |
| CRAB7 | AMK | 4 | 2 | 1 | Additive effect |
|  | ERV | 0.125 | 0.0625 |  |  |
| CRAB8 | AMK | 8 | 2 | 0.75 | Additive effect |
|  | ERV | 0.0625 | 0.03125 |  |  |

**a.** AMK: Amikacin, CR: Carbapenem-resistant, ERV: Eravacycline, FIC:fractional inhibitory concentration, MIC: Minimum inhibitory concentration.
