# Supplementary material for: Amikacin-eravacycline combination mediates the synergistic elimination of carbapenem-resistant pathogens via in vitro and in vivo metabolic reprogramming
Source: PLoS Pathog. 2026 Feb 10;22(2):e1013938. doi: 10.1371/journal.ppat.1013938 (PMC12890146; doi:10.1371/journal.ppat.1013938)
Supplement: S4 Table — (DOCX) [file ppat.1013938.s010.docx]

**S5 Table The functional annotation of hub genes**

| Name | Pathways |
| --- | --- |
| *gltA* | Glyoxylate and dicarboxylate metabolism; TCA cycle |
| *aldA* | Glyoxylate and dicarboxylate metabolism; Pyruvate metabolism |
| *pflB* | Butanoate metabolism; Propanoate metabolism; Pyruvate metabolism |
| *pgi* | Amino sugar and nucleotide sugar metabolism; Starch and sucrose metabolism; Pentose phosphate pathway; Glycolysis / Gluconeogenesis |
| *aceE* | Pyruvate metabolism; TCA cycle; Glycolysis / Gluconeogenesis |
| *pykA* | Pyruvate metabolism; Glycolysis / Gluconeogenesis |
| *tuf2* | Plant-pathogen interaction |
| *rplF* | Ribosome |
| *atpA* | Photosynthesis; Oxidative phosphorylation |
| *rpsD* | Ribosome |
