# Supplementary material for: Amikacin-eravacycline combination mediates the synergistic elimination of carbapenem-resistant pathogens via in vitro and in vivo metabolic reprogramming
Source: PLoS Pathog. 2026 Feb 10;22(2):e1013938. doi: 10.1371/journal.ppat.1013938 (PMC12890146; doi:10.1371/journal.ppat.1013938)
Supplement: S5 Table — (DOCX) [file ppat.1013938.s011.docx]

**Pathological staining**

The experiments were performed by Wuhan servicebio technology CO., LTD.

*Paraffin section making*: Fresh tissue is put into tissue fixative for over 24 h, and stored at room temperature. Remove the tissue from the fixative and smooth it with a scalpel. Place the cut tissue in a dehydration box, then into a dehydrator to dehydrate using gradient alcohol, followed by wax leaching. Melted wax is poured into an embedding frame; prior to solidification, insert the tissue according to embedding surface requirements. Cool at -20°C platform until the wax solidified, then remove and trim the wax block. Slice the trimmed block using a paraffin slicer to obtain 4 μM thick sections. The slice is flattened on warm water from a spreading machine, picked up with glass slide, and baked in an oven. After the water-baked dried wax is melted, it is taken out and stored at room temperature.

*H&E staining*: The paraffin sections are dewaxed using environment friendly dewaxing transparent liquid, and rinsed with tap water. They are then treated with HD constant staining pretreatment solution, followed by hematoxylin staining and eosin staining. The sections undergo dehydration through a series of absolute ethanol (I, II, III), normal butanol (I, II), and xylene (Ⅰ, Ⅱ) before being sealed with neutral gum. Microscopic inspection is performed for image acquisition and analysis. The nucleus appears blue while the cytoplasm is red.

*Immunohistochemistry*: Following dewaxing and hydration, the tissue sections undergo antigen repair and are treated with 3% hydrogen peroxide to block endogenous peroxidase. Subsequently, they are incubated with 3% bovine serum albumin for serum closure. Excess sealing solution is gently shaken off, after which PBS mixed with a specific proportion of primary antibody is added. The sections are then placed flat in a wet box at 4 °C for overnight incubation. After incubation, the slides are washed 3 times with PBS, and covered with the corresponding secondary antibody, followed by incubation at room temperature. After 3 washes with PBS, DAB color developing solution is added. The sections are rinsed with tap water to terminate the color development before undergoing hematoxylin staining for nuclei. The slices are dehydrated through a series of alcohols (75% - 85% - anhydrous ethanol) and n-butanol prior to being cleared in xylene. After slight drying from xylene, the slices are sealed with glue. The results are examined under a white light microscope; hematoxylin-stained nuclei appear blue while positive DAB signals exhibit brown-yellow.
