# Supplementary material for: Amikacin-eravacycline combination mediates the synergistic elimination of carbapenem-resistant pathogens via in vitro and in vivo metabolic reprogramming
Source: PLoS Pathog. 2026 Feb 10;22(2):e1013938. doi: 10.1371/journal.ppat.1013938 (PMC12890146; doi:10.1371/journal.ppat.1013938)
Supplement: S1 Text — (DOCX) [file ppat.1013938.s012.docx]

**Transcriptional profile**

*Experimental method*: BW25113 strains treated with sublethal concentrations of antibiotic were harvested, and total RNA was isolated using the Trizol Reagent (Invitrogen Life Technologies). Quality and integrity were determined using a NanoDrop spectrophotometer (Thermo Scientific) and a Bioanalyzer 2100 system (Agilent). Zymo-Seq RiboFree Total RNA Library Kit was used to remove rRNA from total RNA. Random oligonucleotides and SuperScript III were used to synthesize the first strand cDNA. Second strand cDNA synthesis was subsequently performed using DNA Polymerase I and RNase H (Strand-specific RNA-seq：Then use RNaseH to degrade the RNA strand, and in the DNA polymerase I system, use dNTP with dUTP instead of dTTP as raw material to synthesize the second strand of cDNA). Remaining overhangs were converted into blunt ends via exonuclease/polymerase activities and the enzymes were removed. After adenylation of the 3′ ends of the DNA fragments, Illumina PE adapter oligonucleotides were ligated to prepare for hybridization. To select cDNA fragments of the preferred 400-500 bp in length, the library fragments were purified using the AMPure XP system (Beckman Coulter, Beverly, CA, USA). DNA fragments with ligated adaptor molecules on both ends were selectively enriched using Illumina PCR Primer Cocktail in a 15-cycle PCR reaction. Products were purified (AMPure XP system) and quantified using the Agilent high-sensitivity DNA assay on a Bioanalyzer 2100 system (Agilent). The sequencing library was then sequenced on NovaSeq 6000 platform (Illumina) by Shanghai Personal Biotechnology Cp. Ltd.

*Transcriptome analysis flow*: (1) Data quality control: The quality information of raw data in FASTQ format was calculated and then the raw data was filtered using fastp (0.22.0) software, clean data was obtained by removing reads containing adapter, reads containing ploy-N and low-quality reads. All the subsequent analysis was based on high quality clean data. (2) Data mapping analysis: The reference genome and gene annotation files were downloaded from genome website. Reference genome index was built by Bowtie 2 (2.4.1) and the filtered reads were mapping to the reference genome using Bowtie 2. (3) Expression level analysis: The gene read count value was counted using HTSeq (v 0.9.1) as the original expression level of the gene. In order to make the gene expression levels of different genes and different samples comparable, FPKM (fragments per kilobase of exon per million fragments mapped) is used to normalize the expression. (4) Differential expression analysis: Then difference expression of genes was analyzed by DESeq2 (v 1.38.3) with screened conditions as follows: expression difference multiple |FoldChange| > 1.5, significant P-value < 0.05. At the same time, we used ComplexHeatmap (v 2.16.0) software package to perform bi-directional clustering analysis of all different genes of samples. We got heatmap according to the expression level of the same gene in different samples and the expression patterns of different genes in the same sample with Euclidean method to calculate the distance and Complete Linkage method to cluster. (4.1) Interaction Analysis of Differential Gene Protein Network: The STRING database (https://string-db.org/) is used for protein interaction analysis to reveal the relationship between target genes. (5) Enrichment analysis: We mapped all the genes to Terms in the Gene Ontology database and calculated the numbers of differentially enriched genes in each Term. Using ClusterProfiler (v 4.6.0) to perform GO enrichment analysis on the differential genes (all DEGs / up DEGs / down DEGs), calculate P-value by hypergeometric distribution method (the standard of significant enrichment is P-value <0.05), and find the GO term with significantly enriched differential genes to determine the main biological functions performed by differential genes. ClusterProfiler (v 4.6.0) software was used to carry out the enrichment analysis of the KEGG pathway of differential genes, focusing on the significant enrichment pathway with P-value <0.05.
