# Supplementary material for: Amikacin-eravacycline combination mediates the synergistic elimination of carbapenem-resistant pathogens via in vitro and in vivo metabolic reprogramming
Source: PLoS Pathog. 2026 Feb 10;22(2):e1013938. doi: 10.1371/journal.ppat.1013938 (PMC12890146; doi:10.1371/journal.ppat.1013938)
Supplement: S2 Text — (DOCX) [file ppat.1013938.s013.docx]

**Metabolomics workflow**

This experiment employed GC-MS in accordance with the Metabolomics Standards Initiative (MSI) guidelines throughout the workflow, encompassing sample pretreatment, mass spectrometry detection, data preprocessing, metabolite identification, and bioinformatics analysis[1-7]. (1) Sample pretreatment: A ninefold volume of cold methanol was added to each sample and vortex mixed for 1 min. The mixture was centrifuged, and the supernatant was transferred to a 1.5 mL tube containing an internal standard (0.1 mg/mL ribitol). The supernatant was then dried in a vacuum centrifuge and underwent derivatization through methoximation and trimethylsilylation. (2) GC-MS detection: The metabolic profiles of mouse serum were analyzed using the Agilent 8890-7000E triple quadrupole GC-MS system with an HP-5 ms column. The temperature ramp program is as follows: holding at 85 °C for 3 min; increasing to 280 °C at 7 °C/min for 5 min; then rising at 15 °C/min to 300 °C for 3 min. Helium serves as the carrier gas with a flow rate of 1 mL/min, with mass spectrometry scanning from m/z range of 30-500. (3) Data preprocessing: Mass spectrometry data were processed via Qualitative Analysis software (v 10.0), involving noise reduction, baseline calibration, deconvolution, peak alignment and identification, features extraction, and normalization to minimize discrepancies. (4) Metabolite identification: Metabolites were identified using public databases including the National Institute of Standards and Technology (NIST) and the Human Metabolome Database (HMDB). The structures were confirmed by comparing parameters, including retention time, molecular mass, secondary fragmentation spectra, and collision energy. Identified metabolites underwent verification against reference spectra to ensure an identification level of at least Level 2. (5) Bioinformatics analysis：Employs various methodologies, including univariate and multivariate statistical analyses, differential metabolite screening, and pathway enrichment analysis to explore biological differences and underlying mechanisms.

**References**

1. Rattner J, Farshidfar F, Bathe OF. Gas Chromatography-Mass Spectrometry and Analysis of the Serum Metabolomic Profile Through Extraction and Derivatization of Polar Metabolites. Methods Mol Biol. 2019;1928:235-49. Epub 2019/02/07. doi: 10.1007/978-1-4939-9027-6_13. PubMed PMID: 30725459.

2. Dunn WB, Broadhurst D, Begley P, Zelena E, Francis-McIntyre S, Anderson N, et al. Procedures for large-scale metabolic profiling of serum and plasma using gas chromatography and liquid chromatography coupled to mass spectrometry. Nat Protoc. 2011;6(7):1060-83. Epub 2011/07/02. doi: 10.1038/nprot.2011.335. PubMed PMID: 21720319.

3. Fiehn O, Wohlgemuth G, Scholz M, Kind T, Lee DY, Lu Y, et al. Quality control for plant metabolomics: reporting MSI-compliant studies. Plant J. 2008;53(4):691-704. Epub 2008/02/14. doi: 10.1111/j.1365-313X.2007.03387.x. PubMed PMID: 18269577.

4. Cajka T, Fiehn O. Toward Merging Untargeted and Targeted Methods in Mass Spectrometry-Based Metabolomics and Lipidomics. Anal Chem. 2016;88(1):524-45. Epub 2015/12/05. doi: 10.1021/acs.analchem.5b04491. PubMed PMID: 26637011.

5. Fiehn, O, Robertson, D, Griffin, J, van der Werf, M, Nikolau, B, Morrison, N, et al. The metabolomics standards initiative (MSI). Metabolomics. 2007; 3:3.

6. Li G, Xie C, Lu S, Nichols RG, Tian Y, Li L, et al. Intermittent Fasting Promotes White Adipose Browning and Decreases Obesity by Shaping the Gut Microbiota. Cell Metab. 2017;26(5):801. Epub 2017/11/09. doi: 10.1016/j.cmet.2017.10.007. PubMed PMID: 29117546; PubMed Central PMCID: PMCPMC5695033.

7. Xia J, Wishart DS. Web-based inference of biological patterns, functions and pathways from metabolomic data using MetaboAnalyst. Nat Protoc. 2011;6(6):743-60. Epub 2011/06/04. doi: 10.1038/nprot.2011.319. PubMed PMID: 21637195.
