# Supplementary material for: Amikacin-eravacycline combination mediates the synergistic elimination of carbapenem-resistant pathogens via in vitro and in vivo metabolic reprogramming
Source: PLoS Pathog. 2026 Feb 10;22(2):e1013938. doi: 10.1371/journal.ppat.1013938 (PMC12890146; doi:10.1371/journal.ppat.1013938)
Supplement: S3 Text — (DOCX) [file ppat.1013938.s014.docx]

**Measurement of ATP levels**

Intracellular ATP levels were measured using an ATP Assay Kit (Beyotime, S0026). Bacteria exposed to sublethal concentrations of antibiotics for 4 h were adjusted to an OD_600_ of 1.0, lysed, and centrifuged. The resulting supernatants were mixed with the detection working solution in a 96-well plate, and luminescence (relative light units, RLU) was immediately measured using an Infinite E Plex microplate reader (Tecan). ATP levels were normalized to bacterial cell count for data normalization.
