# Supplementary material for: Amikacin-eravacycline combination mediates the synergistic elimination of carbapenem-resistant pathogens via in vitro and in vivo metabolic reprogramming
Source: PLoS Pathog. 2026 Feb 10;22(2):e1013938. doi: 10.1371/journal.ppat.1013938 (PMC12890146; doi:10.1371/journal.ppat.1013938)
Supplement: S4 Text — (DOCX) [file ppat.1013938.s015.docx]

**Measurement of NADH/NAD^+^ ratio**

The NAD^+^/NADH ratio was determined using an assay kit (Beyotime, S0175). After a 4-h antibiotic treatment, bacterial cells were normalized to an OD_600_ of 1.0 and lysed. The lysate was divided into two aliquots: one was heated at 60 ℃ to degrade NAD^+^ for subsequent NADH measurement; while the other was used to determine total NAD (NAD_total_). Absorbance at 450 nm was measured using an Infinite E Plex microplate reader (Tecan). The NAD^+^ concentration was calculated as the difference between NAD_total_ and NADH.
