# Supplementary material for: Amikacin-eravacycline combination mediates the synergistic elimination of carbapenem-resistant pathogens via in vitro and in vivo metabolic reprogramming
Source: PLoS Pathog. 2026 Feb 10;22(2):e1013938. doi: 10.1371/journal.ppat.1013938 (PMC12890146; doi:10.1371/journal.ppat.1013938)
Supplement: S5 Text — (DOCX) [file ppat.1013938.s016.docx]

**PMF determination**

The PMF was determined using the fluorescent probe 3,3’-diethyloxacarbocyanine iodide (DiOC_2_). Cells were exposed to varying concentrations of CCCP and incubated for 6 h at 37 ℃. Following treatment, cells were harvested by centrifugation, washed twice with PBS, and resuspended to a final density of 10^^6^ CFU/mL. A 10 μL aliquot of 3 mM DiOC_2_ was added to 1 mL of the bacterial suspension, and the mixture was incubated in the dark at 37 ℃ for 30 min. Stained cells were then analyzed by flow cytometry. Red fluorescence and green fluorescence intensities were measured, and the PMF was determined using the formula: log(10^3/2^*(red fluorescence/green fluorescence)) [1]. Data are expressed as the percentage change relative to the untreated control group.

**References**

1. Fang D, Xu T, Li F, Sun Y, Sun J, Yin Y, et al. Methionine-driven methylation modification overcomes plasmid-mediated high-level tigecycline resistance. Nat Commun. 2025;16(1):417. Epub 2025/01/07. doi: 10.1038/s41467-024-55791-w. PubMed PMID: 39762254; PubMed Central PMCID: PMCPMC11704046.
